# Circular RNA expression profile of spleen in a *Clostridium perfringens* type C‐induced piglet model of necrotizing enteritis

**DOI:** 10.1002/2211-5463.12512

**Published:** 2018-09-17

**Authors:** Zunqiang Yan, Tiantuan Jiang, Pengfei Wang, Xiaoyu Huang, Qiaoli Yang, Wenyang Sun, Shuangbao Gun

**Affiliations:** ^1^ College of Animal Science and Technology Gansu Agricultural University Lanzhou China; ^2^ Gansu Research Center for Swine Production Engineering and Technology Lanzhou China

**Keywords:** circRNAs, *Clostridium perfringens* type C, intestine, necrotizing enteritis, piglets, spleen

## Abstract

*Clostridium perfringens* type C is a pathogen that causes necrotizing enteritis (NE), which is an intestinal tract disease in piglets. The pathogenesis of *C. perfringens* type C‐induced NE is still unclear, leading to a lack of effective therapies. Earlier studies have reported that circular RNAs (circRNAs) are involved in the pathogenic processes of various diseases. However, it is not known if circRNAs in spleen play a role in *C. perfringens* type C infection in NE. To address this question, we infected 7‐day‐old piglets with *C. perfringens* type C to induce NE. Hematoxylin and eosin staining of small intestine revealed inflammation, atrophy and shedding of intestinal villi, and intestinal mucosal necrosis. We observed increased expression of cytokine genes (such as *IL‐1β* and *IL‐6*) and inflammation in the spleen. In addition, we used RNA‐seq and bioinformatics analysis to examine changes in circRNA expression. A total of 103 circRNAs were found to be differentially expressed in NE, and Gene Ontology analysis revealed that the genes producing differentially expressed circRNAs were enriched in regulation of the cellular metabolic process protein binding. Kyoto Encyclopedia of Genes and Genomes pathway analysis showed that the genes producing differentially expressed circRNAs were involved in the tumor necrosis factor signaling pathway, T cell receptor signaling pathway and nuclear factor‐κB signaling pathway. Finally, we found eight circRNAs (including circ_0002220 and circ_0000821) that are related to NE. Therefore, our study provides new insights into the mechanisms underlying *C. perfringens* type C infection in piglets.

**Abbreviations**
circRNA: circular RNA
CS: control group's spleen
GO: Gene Ontology
KEGG: Kyoto Encyclopedia of Genes and Genomes
NE: necrotizing enteritis
NF‐κB: nuclear factor‐κB
RT‐qPCR: real time‐quantitative PCR
TNF: tumor necrosis factor
TPM: transcripts per kilobase million
TS: treated group's spleen

Circular RNAs (circRNAs) are non‐coding RNAs that are present in various species, such as archaea 1, human 2 and zebrafish 3. Compared with linear RNAs, circRNAs are characterized as highly stable in tissues and can be released into the extracellular space via the exosomes 4, 5. CircRNAs are covalently closed, single‐stranded transcripts that are generated from pre‐mRNA back‐splicing in exons, introns or intergenic regions and do not have a 5′ end cap and 3′ poly(A) tail 6. CircRNAs are receiving more attention, as they can act as miRNA sponges to regulate gene expression in physiological and pathological processes 7. Moreover, circRNAs play important roles in pathogenesis of various infectious diseases, such as tuberculosis 8, hemorrhagic fever 9, avian leukosis virus‐induced tumor 10, and grass carp hemorrhagic disease 11. Huang *et al*. 8 revealed that up‐regulated hsa_circ_0043497 and down‐regulated hsa_circ_0001204 may be diagnostic biomarkers for tuberculosis caused by *Mycobacterium* *tuberculosis*. Wang *et al*. 9 showed that up‐regulated circRNA‐chr19 targets miR‐30b‐3p to regulate *CLDN18* expression under the stimulation of Ebola virus reproduction. In addition, circRNAs are potential candidates as diagnostic molecular biomarkers in some diseases. Chen *et al*. and Tian *et al*. revealed that hsa_circ_0003159 and circPVT1 are potential prognostic marker in gastric cancer 12, 13. Zhang *et al*. 14 suggested that hsa_circ_0014130 may be a biomarker in non‐small cell lung cancer.

*Clostridium perfringens* type C leads to common digestive tract disease in most mammalian species, especially piglets 15, 16, which is characterized by inflammation of the small intestine, destruction of muscle, multiple organ failure, and even death in human and animals 16, 17. In some patients and animals, *C. perfringens* type C infection progresses so quickly that death precedes diagnosis, because *C. perfringens* type C can evade and impair innate immunity in the host 18. To establish effective strategies, it is essential to understand how immune cells respond to infection and how this response changes during infection. However, the host–*C. perfringens* type C interaction is poorly understood, and knowledge of the mechanism underlying *C. perfringens* type C infection is still limited.

In this study, we established a necrotizing enteritis (NE) model in piglets treated by *C. perfringens* type C. We chose the spleen as the target tissue to explore the circRNA profile and the potential functions of circRNAs in NE, because spleen is considered to be the most important immune organ in host immune defense against several bacterial pathogens 19, 20. Combining our output data with the public *Sus scrofa* genome dataset, we found many novel circRNAs that may help improve our knowledge of the pathogenesis of NE in piglets.

## Materials and methods

### Ethics statement

This experiment was approved by the ethics committee of College of Animal Science and Technology, Gansu Agricultural University (approval number 2006‐398).

### *Clostridium perfringens* type C growth condition

*Clostridium perfringens* type C strain (CVCC 2032) was purchased from China Veterinary Culture Collection Center. It was cultured in bouillon culture medium (HopeBio, Qingdao, China) for reproduction at 37 °C for 16 h. The colony‐forming units (CFUs) were evaluated by yolk plate colony counting (HopeBio). For preparation of the yolk plate, the following was used: 20 mL 50% egg yolk + 11.75 g tryptose sulfite cycloserine agar base + 20 mL 0.5% d‐cycloserine + 250 mL distilled water. Ten‐fold serial dilutions were performed on the yolk plate for *C. perfringens* type C and incubated at 37 °C for 24 h. According to the count result, the density of bacterium was adjusted to 1 × 10^9^ CFUs·mL^−1^.

### *Clostridium perfringens* type C‐induced piglets NE

To avoid the maternal antibodies, we detected the serum from a sow and selected the negative sow's piglets to conduct the experiment. In our study, 7‐day‐old healthy piglets were the descendants (Y × L) from the nucleus herd in Dingxi city, Gansu province. These piglets had no diarrhea and no infection with *C. perfringens*,* Escherichia coli* and *Salmonella* as determined with commercial ELISA kits (Jiancheng Bioengineering Institute, Nanjing, China). *C. perfringens* type C directs to α toxin and monoclonal antibody of α toxin. *E. coli* directs to K88 and monoclonal antibody of K88. *Salmonella* directs to O7 and monoclonal antibody of O7. In order to obtain serum, blood samples were collected via the anterior vena cava, placed in sterile tubes for natural coagulation, and centrifuged at 2000 ***g*** for 10 min at 4 °C.

All piglets had free access to antibiotic‐free feed and water throughout the trial. To avoid cross‐infection, each piglet was housed in single cages with appropriate conditions of climate control at the animal testing ground. Three piglets were randomly selected as the treated group (TS, treated group's spleen). Each piglet was dosed with 1 mL of bouillon culture‐medium containing *C. perfringens* type C by oral gavage and treated once a day for 5 days. The remaining three piglets were selected as the control group (CS, control group's spleen) and each piglet received an equal volume of sterile medium. During the period of the 5‐day experiment, we recorded diarrheal times and fecal state of each time. Fecal symptom traits (0 = normal, solid feces; 1 = slight diarrhea, soft and loose feces; 2 = moderate diarrhea, semi‐liquid feces; 3 = severe diarrhea, liquid and unformed feces) were evaluated by a previously described method 21, 22. Student's *t* test was used to judge significance of total scores between CS and TS.

### Sample collection

Piglets were humanely sacrificed as necessary to ameliorate suffering. Piglets were euthanized using 10 mg kg^‐1^ ketamine hydrochloride by intramuscular injection. We complied with the regulations in *Establishment and Application of Small Pig Medical Research Model* (Chen Hua (ed), People's Medical Publishing House, pp. 66–73). Six piglets were humanely euthanized at 5 days post‐infection; each spleen was flushed with PBS, frozen in liquid nitrogen and stored at −80 °C until RNA extraction.

### RNA isolation and quality control

Total RNA samples were extracted from each spleen using Trizol reagent (Invitrogen, Waltham, MA, USA). RNA quality was estimated by the Agilent Bioanalyzer 2100 (Agilent Technologies, Santa Clara, CA, USA). The quantification of RNA was assessed using a Nanodrop 2000 (Thermo Scientific, Waltham, MA, USA). The integrity of RNA was assessed by using the 1% formaldehyde denaturing gel electrophoresis.

### CircRNA sequencing

According to the Epicentre Ribo‐zero™ rRNA Removal Kit (Epicentre, San Diego, CA, USA) instruction, total RNA (3 μg) per sample was treated to remove rRNAs. Then, the rRNA‐depleted RNA was treated with RNase R to digested linear RNAs. Residual RNA was used to generate circRNA libraries using NEBNext^®^ Ultra™ Directional RNA Library Prep Kit for Illumina^®^ (New England Biolabs, Ipswich, MA, USA). Library sequencing was conducted on an Illumina HiSeq 4000 instrument with 150‐bp paired‐end reads at the Beijing Novogene Bioinformatics Institute in China.

### CircRNA identification and quantification

Clean reads were obtained from raw reads and then mapped to the pig reference genome (Sscrofa 10.2) using bowtie2 23. Reads that aligned contiguously to the pig genomes were removed. From the remaining reads, the potential circRNAs were detected and identified using find_circ 2 and ciri2 24. The raw counts of potential circRNAs were first normalized using transcripts per kilobase million (TPM) to assess expression levels 25.

### Differential expression analysis

Differential expression analysis was performed using the deseq2 26. The resulting *P* values were adjusted to control the false discovery rate. CircRNAs (adjusted *P* value < 0.05) were assigned as differentially expressed.

### RT‐qPCR validation

Six circRNA and eight cytokine genes were selected to evaluate expression levels by real time‐quantitative PCR (RT‐qPCR). Total RNAs were reverse transcribed into cDNA using a PrimeScript™ RT Reagent kit (Takara, Dalian, China). RT‐qPCR assays were conducted using the SYBR^®^ Green PCR Master Mix (Takara) in a Roche LightCycler 480II instrument (Roche Applied Science, Penzberg, Germany). The dissociation curve was used to estimate the specificity of PCR products. The expression level of circRNAs were normalized to β‐actin (internal standard control) and calculated using the $2^{-\Delta \Delta C_{t}}$ method 27. The significance of gene expression levels was evaluated by Student's *t* test between CS and TS.

### GO and KEGG pathway analyses

Gene Ontology (GO) enrichment analysis for parent genes of differentially expressed circRNAs was performed by goseq r 28. The related Kyoto Encyclopedia of Genes and Genomes (KEGG) pathways of the host genes of differentially expressed circRNAs were analyzed with kobas 29.

### Prediction of circRNA–miRNA interactions

Differently expressed circRNAs were selected for prediction of circRNA–miRNA interactions, which were identified by miranda. Then, a circRNA–miRNA interaction network was generated by cytoscape 30.

### Histopathology of the spleen and small intestine

Small intestine and spleen were fixed in 10% buffered formalin for 24 h. Then these samples tissues were processed for histology and stained with hematoxylin and eosin.

## Results

### Fecal scores, cytokines expression and histological damage between TS and CS

**Figure 1:**
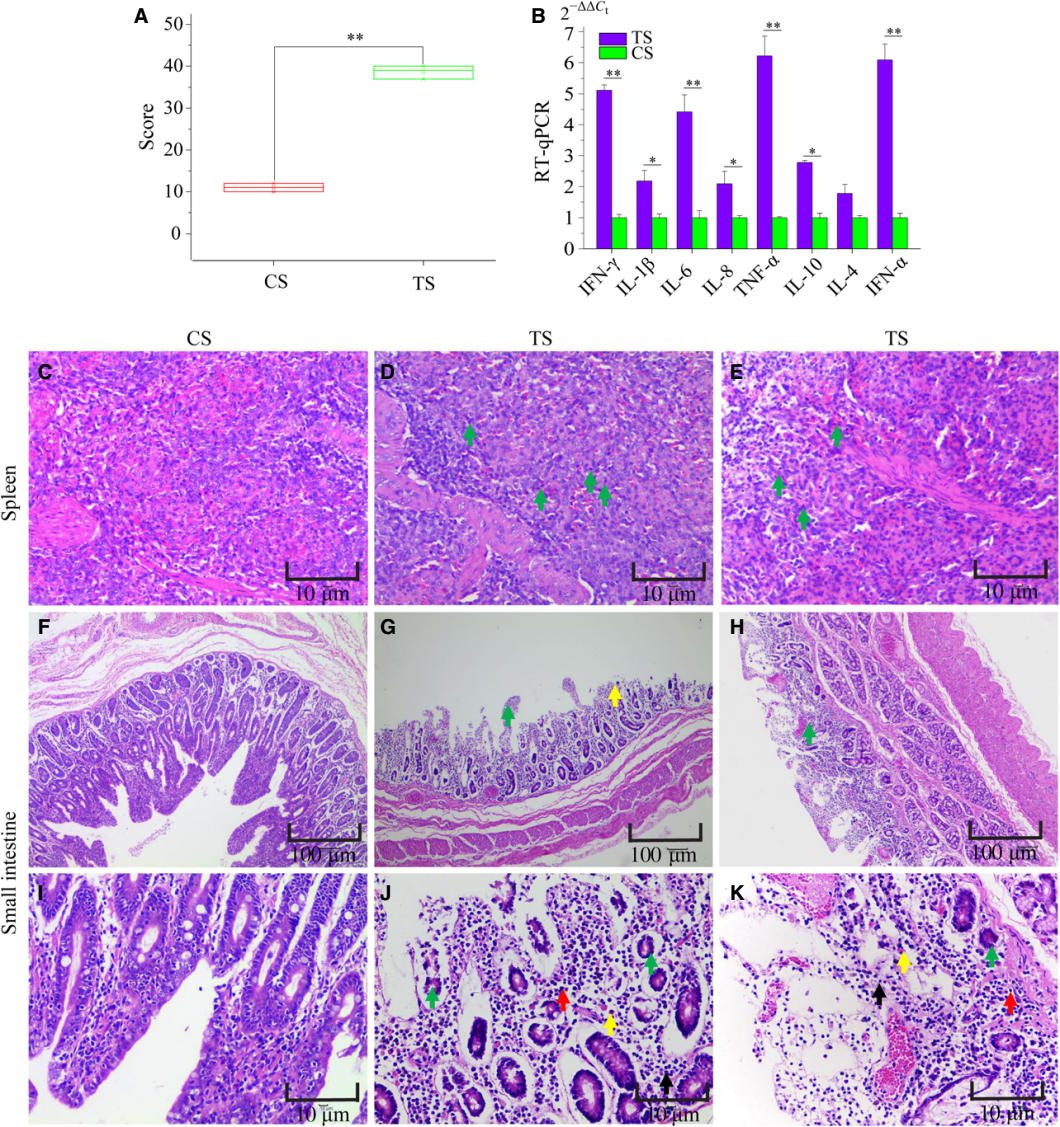
(A) The total fecal scores during the period of 5 days. (B) Relative expression level of cytokines in spleen (mean ± SE). IFN, interferon; IL, interleukin. (C) Normal spleen (scale bar: 10 μm). (D, E) Infiltration with moderate amounts of neutrophilic granulocytes (green arrows) (scale bar: 10 μm). (F) Normal small intestine (scale bar: 100 μm). (G) Intestinal villus atrophy (green arrow) and shedding (yellow arrow) (scale bar: 100 μm). (H) Intestinal mucosal necrosis (green arrow) (scale bar: 100 μm). (I) Normal small intestine (scale bar: 10 μm). (J, K) Intestinal gland atrophy (green arrow), macrophagocyte infiltration (yellow arrow), plasmocyte infiltration (red arrow) and neutrophil granulocyte (black arrow) (scale bar: 10 μm). Asterisk above bars indicate significant differences (*P <* *0.05, **P <* *0.01). Student's *t* test was used to judge significance of total scores and relative expression level between CS and TS.

We compared the total fecal scores, the expression of cytokine genes and histological damage of spleen and small intestine between the TS and CS groups. Piglets in the TS group were characterized by persistent diarrhea and had higher feces scores than piglets in the CS group (Fig. 1A). These cytokine genes were significantly differentially expressed between the TS and CS groups (Fig. 1B). The spleen of CS piglets was normal (Fig. 1C), but the spleen of TS piglets showed infiltration with moderate amounts of neutrophilic granulocytes apparent in the red pulp (Fig. 1D,E). The small intestine of CS piglets was normal (Fig. 1F,I). TS piglets had intestinal lesions characterized by intestinal villus atrophy and shedding, intestinal gland atrophy, intestinal mucosal necrosis and abundant inflammatory cell infiltration (Fig. 1G,H,J,K).

### Identification and characterization of circRNAs in spleen

**Figure 2:**
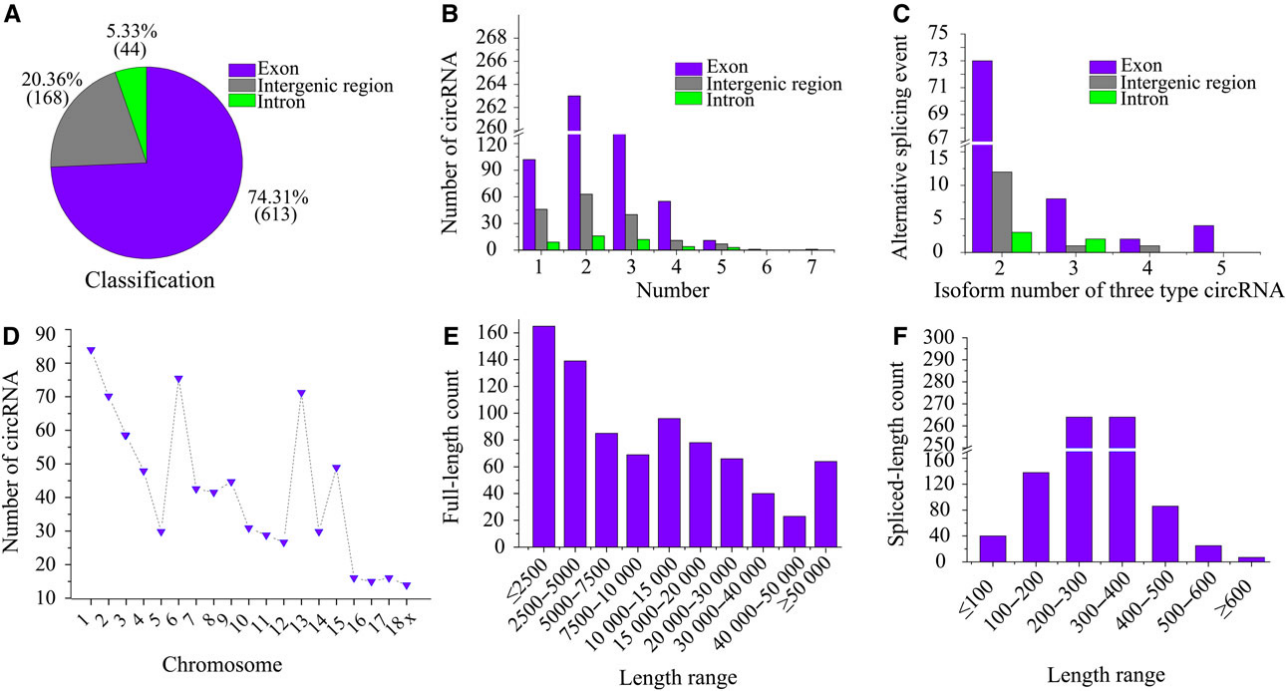
(A) Classification of expressed circRNAs in the spleen based on genomic origins. (B) Number of circRNAs with the indicated number of exons, intergenic regions and introns. (C) Summary of alternative splicing events. (D) The count of circRNAs detected in pig chromosomes. (E) The number of circRNAs in different full‐length ranges. (F) The number of circRNAs in different spliced‐length ranges.

Overall, a total of 825 circRNAs were found in pairs of CS and TS samples (Table S1). Among them, a mass of circRNAs originated from protein‐coding exons, accounting for 74.31%. Some were from introns, and a few were from intergenic regions (Fig. 2A). We found that a majority of circRNAs had two exons, or introns or intergenic regions. One exonic circRNA had six exons, and one intergenic circRNA had seven intergenic regions (Fig. 2B). We obtained 106 (87 from exon regions, 14 from intergenic regions and five from intron regions) alternative back‐splicing circularization events. The majority of chromosome loci generated a single circular transcript. In addition, 88 of 106 events produced two different circRNAs isoforms, 11 produced three distinct isoforms, three produced four isoforms, four produced five isoforms (Fig. 2C). Regarding chromosome distribution, chromosome 1 has most circRNAs, followed by 6 and 13 (Fig. 2D). The size of the circRNAs ranged from less than 2.5 kb to greater than 50 kb. The majority of circRNAs are shorter than 20 kb (Fig. 2E). Most circRNAs are shorter than 400 nt. The average length was 230 nt, while the maximum length was 713 nt, and the minimum length was 22 nt (Fig. 2F).

### Differentially expressed circRNAs from the sequencing profile

**Figure 3:**
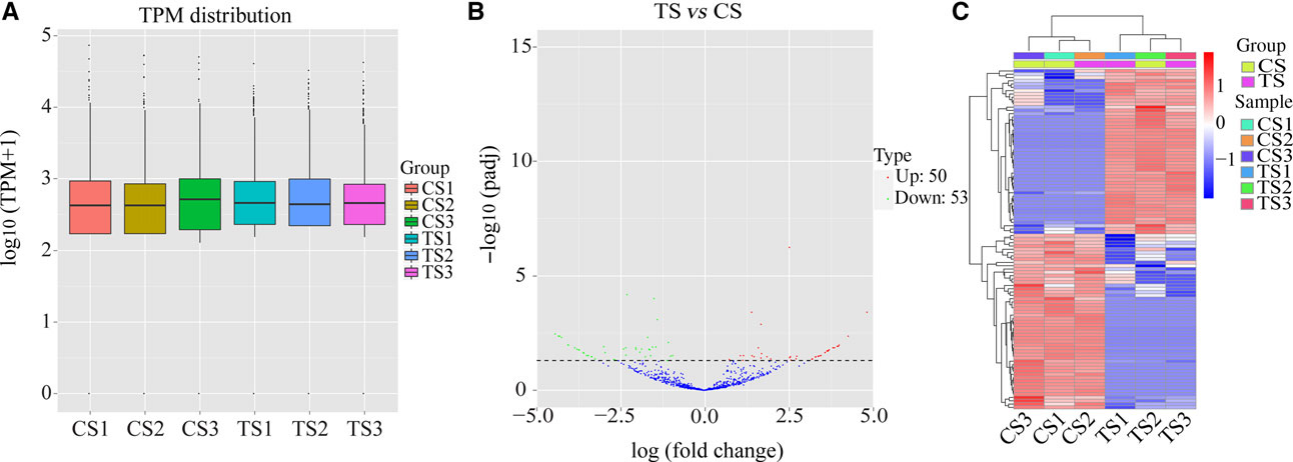
(A) Expression level indicated by log10 (TPM + 1) in the CS and TS circRNAs. (B) Volcano plots showing the differential expression of the circRNAs (*P *<* *0.05). The red and blue points in the plot represent the differentially up‐ or down‐regulated circRNAs, respectively. (C) Clustered heatmap of the differentially expressed circRNAs in paired CS and TS samples. Rows represent circRNAs and columns represent different treated tissues.

Distribution of the circRNA expression profiles in all samples was measured based on TPM, and was not different (Fig. 3A). To explore whether circRNAs have biological functions in spleen of piglet after *C. perfringens* type C infection, we compared the expression profiles of differentially expressed circRNAs between CS and TS sample. Volcano plot showed that 103 circRNAs were differentially expressed between the two groups (*P *<* *0.05), including 50 up‐regulated circRNAs and 53 down‐regulated circRNAs (Fig. 3B). A clustered heatmap was constructed to reveal distinguishable circRNA expression profiling of the samples. The result showed that the expression levels of circRNAs were variable between the CS and TS groups (Fig. 3C).

### Validation of differentially expressed circRNAs

**Figure 4:**
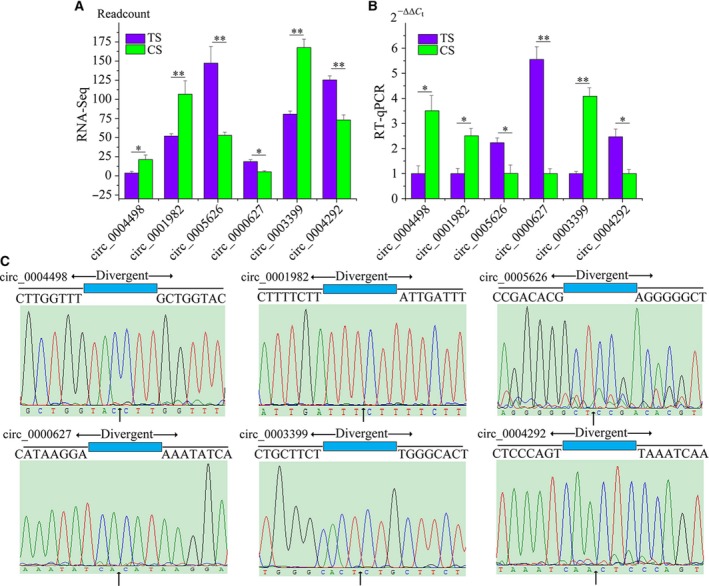
(A) RNA‐Seq results. (B) RT‐qPCR results. The results were presented as the mean ± SE of three replicates (*P <* *0.05; **P <* *0.01). (C) Validation of the head‐to‐tail back‐splicing of circRNAs by Sanger sequencing (vertical arrow represents head‐to‐tail site of circRNAs).

Six randomly selected circRNAs were chosen for further validation with divergent primers by RT‐qPCR (Table S2). Products used were confirmed by Sanger sequencing. RT‐qPCR results were consistent with the sequencing results (Fig. 4A,B). The results showed that these circRNA sequences had covalently closed, continuous loop structures, which is consistent with prediction (Fig. 4C). Therefore, our circRNA profile was reliable.

### GO and KEGG analyses of the parental genes of circRNAs

**Figure 5:**
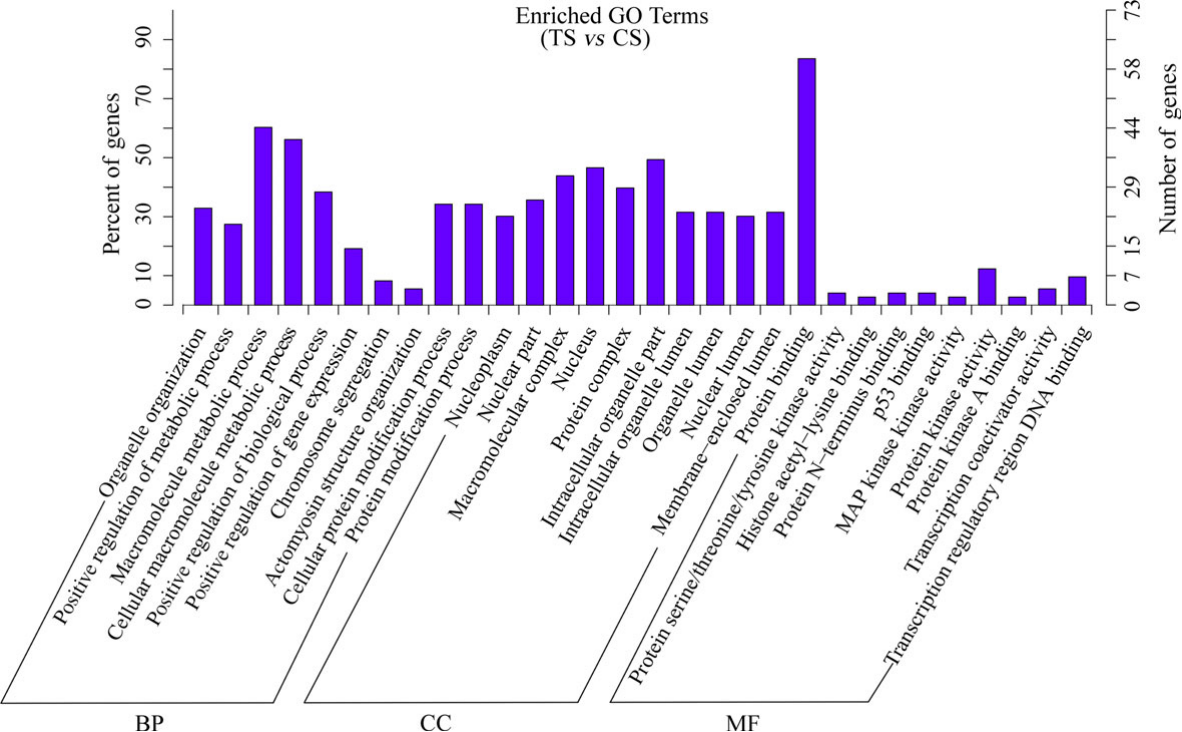
GO analysis of the biological functions of the differential circRNAs according to parent genes annotations. BP, biological process; CC, cellular component; MF, molecular function.

**Figure 6:**
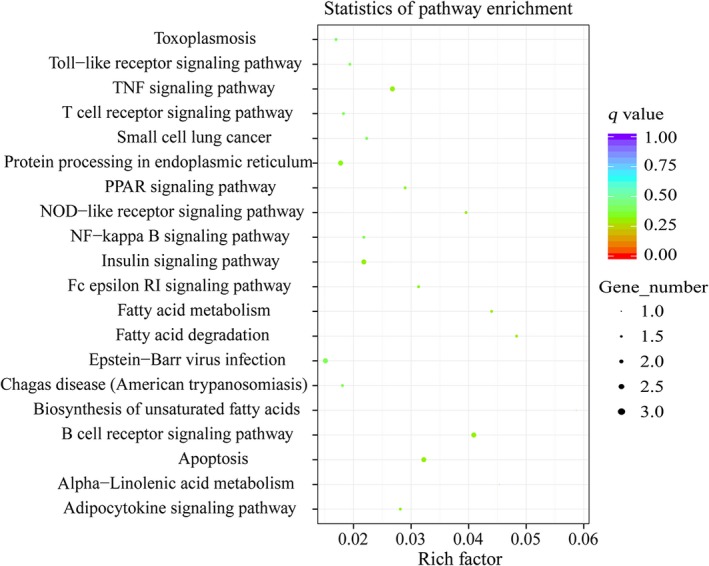
KEGG pathways of the differential circRNA parental genes. Rich factor is the ratio of the number of genes located in the KEGG pathway to the total number of genes in the KEGG pathway. The *q*‐value represents the *P*‐value after the multiple hypothesis test correction.

To explore the function of differentially expressed circRNAs, we predicted and annotated their parent genes. Gene ontology was analyzed for three different aspects, namely biological process, cellular component and molecular function. The top 30 enrichment GO terms are shown in Fig. 5. For the biological process group, the most enriched and meaningful GO terms were associated with cell metabolism and regulation, such as macromolecule metabolic process and cellular macromolecule metabolic process. For the cellular components, the main represented GO terms were intracellular organelle part, nucleus. For molecular function, the main represented category was protein binding. KEGG pathway analysis revealed that a total of 74 pathways were obtained. Among these KEGG pathways, the top 20 pathways are listed in Fig. 6. Some of them are associated with immune system function. The most enriched and meaningful pathways were related to tumor necrosis factor (TNF) signaling pathway, B cell receptor signaling pathway, T cell receptor signaling pathway and nuclear factor‐κB (NF‐κB) signaling pathway.

### Prediction of circRNAs/miRNAs interaction

**Figure 7:**
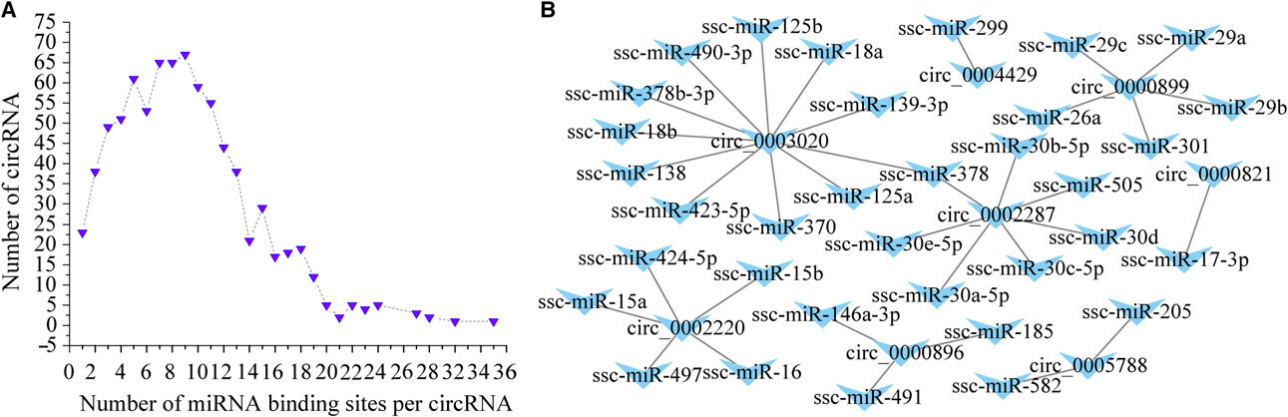
(A) Number of miRNA‐binding sites per circRNA. (B) CircRNA–miRNA network constructed and visualized.

In the present study, 812 of 825 circRNAs had miRNA‐binding sites. On average, each circRNA could bind nine miRNAs. Most circRNAs (67) had nine miRNA‐binding sites. One circrRNA (novel_circ_0002919) had 33 miRNA‐binding sites (Fig. 7A).

Three criteria were used to screen the potential circRNA–miRNA network involved in NE. Firstly, the miRNAs that predicted by dysregulated circRNAs should be high homology (searching for the miRNAs in miRBase; Table S3‐1). Secondly, the miRNAs that were predicted by dysregulated circRNAs should be associated with immune response of infectious disease (searching for the miRNA in Web of Science; Table S3‐2). Thirdly, parent genes of dysregulated circRNAs should be involved in immune response‐related KEGG pathways, such as the TNF signaling pathway and B cell receptor signaling pathway (Table S3‐3). Finally, a total of 34 miRNAs were found to be targets of eight circRNAs (Fig. 7B and Table S3‐4).

## Discussion

The host immune responses are activated after animals are infected by *C. perfringens*. Previous studies have found that immune‐related genes (such as *IL‐1β* and *IL‐6*) are up‐regulated in the spleen of NE‐infected broilers 31. Our results also showed that *IL‐1β* (*P *<* *0.05) and *IL‐6* (*P *<* *0.01) are increased in the spleen of TS compared with CS piglets (Fig. 1B). One study found that infiltration with neutrophilic granulocytes occurred in the small intestine of piglets with NE in natural *C. perfringens* type C infection 32. Indeed, there are obvious pathological changes (such as inflammation, intestinal villus atrophy and shedding and intestinal mucosal necrosis) in the small intestine (Fig. 1G–H,J–K) of TS piglets. These results suggested that *C. perfringens* type C caused piglet NE in our study. Among the 825 circRNAs identified, we found 103 were differentially expressed (50 up‐regulated and 53 down‐regulated) between the TS and CS groups, indicating their special roles in resisting *C. perfringens* type C (Fig. 3B). The study demonstrated that the expression level of most circRNAs and their parent genes was significantly positively correlated, which helps us to understand the function of circRNAs from their parent genes 33. In this study, GO and KEGG pathway analyses were conducted to better explore the parental gene function of these differentially expressed circRNAs. These parental genes are enriched in immune system function‐related GO terms and KEGG pathways, such as p53 binding, MAP kinase kinase activity, Toll‐like receptor signaling pathway and the NF‐κB signaling pathway (Figs 5 and 6). Indeed, previous studies also found that the differentially expressed genes in the spleen of NE‐afflicted chicken caused by *C. perfringens* type A were enriched in these terms and pathways 20, 34. Hence, we speculated that circRNA alterations were related to the spleen of *C. perfringens* type C‐induced NE model.

circRNAs have been reported to act as natural miRNA sponges via competitive binding to microRNA response elements 7. Combined with the sponge theory, we constructed a circRNA–miRNA regulation network, which helped us to better understand the functional importance of circRNAs. We screened eight circRNAs that contained miRNA‐binding sites, suggesting that these circRNAs may function as miRNA sponges in NE. Target prediction shows 34 miRNAs to be the targets of these eight circRNAs (Fig. 7B). Of these, circ_0002220 targets miR‐15b, which is associated with *Salmonella* infection 35 and encephalitis virus infection 36, and miR‐16, which can regulate macrophage phagocytosis in bacterial infection. In myeloid cells, deleting miR‐15a/16 (miR‐15a/16^−/−^) can significantly reduce the *E. *coli infection‐associated mortality 37. *IKBKBI*, the parent gene of circ_0002220, is enriched in MAPK signaling pathway, TNF signaling pathway, NF‐κB signaling pathway and Toll‐like receptor signaling pathway. These results suggested that circ_0002220 may play an important role in *C. perfringens* type C infection via miR‐15a/16. Furthermore, circ_0003020 and circ_0002287 commonly target miR‐378, which was down‐regulated in patients infected with dengue virus. Over‐expression of miR‐378 facilitated dengue virus replication in dengue virus‐infected mice 38. *RBL1*, the parent gene of circ_0002287, is enriched in viral carcinogenesis and transforming growth factor β signaling pathway. These results suggested that circ_0002287 is involved in *C. perfringens* type C infection via miR‐378.

Our study further expands our understanding regarding the regulation of NE. We obtained the circRNA profile of the *C. perfringens* type C‐induced NE model and showed the potential circRNA functions in NE by bioinformatics. However, the precise underlying molecular functions of circRNAs in NE pathogenesis are still unknown. Further work will need to be conducted.

## Author contributions

ZY and TJ performed the experiments and wrote the manuscript. PW, XH and QY analyzed and interpreted the data. WS and SG participated in project design. All authors read and approved the final manuscript.

## Supporting information


**Table S1.** Information for the novel circRNAs.

**Table S2.** Primers for the selected circRNA and cytokine genes.

**Table S3.** miRNA–circRNA network.
